# A Pediatric Case of Accidental Eucalyptus Oil Poisoning from New Delhi, India: Emergency Measures, Historical Context, and Implications for Practice

**DOI:** 10.7759/cureus.5734

**Published:** 2019-09-23

**Authors:** Ramakrishnan Sitaraman, Gangotri Rao

**Affiliations:** 1 Biotechnology, TERI School of Advanced Studies, New Delhi, IND; 2 Pediatrics, Aashlok Hospital, New Delhi, IND

**Keywords:** essential oil, poisoning, essential oil toxicity, eucalyptus oil, accidental ingestion

## Abstract

Eucalyptus oil (EO) and EO containing products are readily available worldwide over the counter as topical nasal decongestants, rubefacients, anti-pyretics, and anti-inflammatory agents. However, EO is poisonous when orally ingested, or otherwise internally administered, resulting in serious outcomes such as seizures, vomiting, drowsiness, and even death. In this case report, we describe emergency measures adopted in a suspected case of EO ingestion by a 17-month-old female infant. It was found that stomach washes with normal saline followed by the oral administration of ranitidine to prevent vomiting restored and maintained normalcy over a 24-hour period. We situate our experience within the Indian context and recommend that pediatricians and healthcare workers routinely and explicitly warn parents about the serious consequences of the incorrect usage of substances containing essential oils in general and EO in particular.

## Introduction

The beneficial effects of eucalyptus oil (EO) either singly or in combination with volatile substances like camphor and menthol, as well as its toxic properties in high doses have been well known for a long time (for a recent review, see reference [1]). This has led to its worldwide usage, either singly or in combination, as a home remedy for its decongestant, rubefacient, anti-inflammatory, analgesic, and antipyretic properties. Additionally, EO is also used in topically applied insect repellents. However, EO also contains toxic compounds including hydrocyanic acid that may cause multiple adverse reactions such as seizures, vomiting, drowsiness, coma and, in sufficiently high doses, lead to death [2]. Moreover, owing precisely to its ready availability and widespread usage in households, there is the ever-present hazard of children swallowing EO or EO-containing formulations. The first case report of EO poisoning in English language that we could trace dates back to 1898 from the former princely state of Travancore, now part of Kerala, India [3]. A second early case report dates back to 1911, this time from the U.K. [4]. Other, more recent case studies are available in the medical literature [5-9]. A retrospective analysis of cases of essential oil ingestion by infants admitted to the The Hospital For Sick Children, Toronto, Canada found that, out of 251 cases recorded during December 1995 to March 1997, EO or formulations containing EO accounted for 233 cases [10]. Likewise, 41 cases of EO ingestion in children under 14 were reported to the Mater Children’s Hospital, South Brisbane, Queensland, Australia between July 1, 1984 and July 30, 1994 [11]. Day *et al.* surveyed poisoning cases from four medical facilities in the state of Victoria, Australia, and noted that poisoning by EO was over-represented in the sample relative to poisoning by other agents [12]. A qualitative study conducted in Cambodia in 2017 noted that the widespread usage of menthol and eucalyptus-derived terpenes in rubbing oils as well as ointments and recommended counseling about the toxic aspects of these products for parents and caregivers [13]. A study of 10 cases of EO poisoning-induced epileptic seizures based on admissions at three tertiary care hospitals located in Bengaluru, India found that the patients had been exposed to EO via inhalation (eight cases), intranasal instillation (one case), and massage (one case) [14]. From some of the available case studies we note that while the maximum number of cases may be attributed to ingestion, even topical application or inhalation of EO vapors can also result in adverse reactions [6-7,14-16]. Intriguingly, the Queensland study also noted that 33 children were entirely asymptomatic, including four who had reportedly swallowed more than 30 mL of EO [11]. Only two children out of these 41 exhibited symptoms requiring some medical attention, but not advanced life support. Thus, it is safe to surmise that, starting with the 1898 case report, the problem of inappropriate exposure to EO continues to recur to the present day in a variety of settings, exhibiting a wide range of clinical outcomes. 

## Case presentation

A female infant aged 17 months was admitted to Aashlok Hospital after having accidentally ingested approximately 0.5 mL of store-bought EO. The child exhibited symptoms of drowsiness but no seizures, and her heart rate and respiratory rates were 140 beats/minute and 40/minute respectively. Ausculation revealed that her lungs were clear and that her S1 and S2 heart sounds were of normal intensity. No murmur was found over the precardium. Abdominal examination by palpation indicated that the liver and spleen were of normal size.

The medical history of the child was unexceptionable, starting with normal delivery after a full term gestation, with a satisfactory immunization record and no known pre-existing conditions. After confirming that she had no known allergies, stomach washes were carried out twice with 50 mL of normal saline followed by aspiration using a feeding tube. One milliliter (25 mg) of the histamine H2-receptor antagonist ranitidine (25 mg) was orally administered to prevent vomiting and consequent dehydration (administration of an anti-emetic before stomach washes is not recommended during initial case presentation because vomiting may actually help eliminate the ingested poison in the early stages).

In spite of this treatment, the child remained drowsy but without any vomiting. Therefore, she was kept under observation overnight, which passed uneventfully. At the time of discharge her heart rate was 130/minute and the respiratory rate stood at 36/minute. A systemic examination was carried out, and no abnormalities were detected. She was alert and active indicating the absence of any lasting adverse effects on the central nervous system. Her parents were advised to report to the hospital in the event of the recurrence of symptoms within 24 hours. However, no adverse event recurred and the child made a full recovery.

## Discussion

The ingestion of household items - household chemicals, medicines, and objects - by children is an avoidable and well-recognized hazard. Based on the medicinal properties of plant-derived essential oils and their seeming innocuousness due to their ‘natural’ origins, the public may be inclined to think that these are safe for usage, including even for internal consumption. Historically, standard pharmacopeias have also stipulated dosages of EO for internal consumption. The case report from 1898 [3] specifically mentions that oral EO administration was recommended in the then-current edition of Whitla’s Pharmacy [17]. The case report of 1911 again mentions the ingestion of EO within recommended dosage limits [4]. Likewise, Patel and Wiggins also note in their 1980 case report of EO ingestion [5] that, as per Martindale: The Extra Pharmacopeia (1977), an oral dose of 0.06-0.2 mL of EO was considered safe [18]. In addition to children swallowing EO, the inadvertent administration of EO to children by adults has been reported. In the survey by Day *et al*. (1997) seven such instances were reported [12]. This happened because the adult either mistook the bottle for another formulation (six cases) or was grossly mistaken about the proper usage of the product (one case). The same survey also pointed out that in most cases access to EO was gained due to its usage in household vaporizer units, rather than direct access to the oil container. Finally, one of us (R.S.) would like to place on record an admission by an elderly individual in 1978 of routine oral self-administration of a topical formulation containing EO, menthol, and methyl salicylate mixed in milk during colds and coughs, but without any adverse outcomes. Owing to the wide range of outcomes observed upon ingestion of EO and the lack of standardization preventing meaningful comparison between different preparations available in the market, it is probably better to avoid ingesting EO and take adequate measures to prevent accidental ingestion. 

Improper usage or overdoses of readily available essential oils and other ‘medicinal’ substances generally regarded as safe are especially important in the context of the large disparities in the availability of qualified medical practitioners in urban versus rural areas of India. This problem was prominently highlighted in a report on the status of the health workforce in India based on data obtained from the 2001 census published by the World Health Organization (WHO) in 2016 [19]. The WHO report noted that 41.6% of 'allopathic' (i.e. conventional modern medicine) practitioners in urban areas and 81.2% in rural areas did not possess any medical qualifications. When viewed on an all-India basis, this amounted to 57.3% of all medical practitioners not having any medical qualification. More troublingly, this implies that even though India has already achieved the WHO-recommended ratio of one qualified doctor per 1000 population [20], there are sizable numbers of unqualified practitioners having active access to patients. This situation is potentially exacerbated by the presence of 3.8 times more doctors in urban areas than in rural ones [19]. Thus, even though there are numerically ‘sufficient’ numbers of qualified doctors in India, their distribution is highly uneven with highest concentrations being found in the large metro cities. Simultaneously, a large fraction of the rural population remains underserved, probably resulting in a greater dependence on self-medication, over-the-counter home remedies, and unqualified practitioners than we realize. Therefore, there is a real danger of poisoning cases of both adults and children by such household remedies remaining unrecognized and under-reported. 

Under these circumstances, pediatricians and general practitioners should try to familiarize themselves with local usage patterns of home remedies and accordingly counsel parents and caregivers about the dangers inherent in their incorrect usage, especially by children. More generally, all health workers (including para-medical and traditional caregivers) could undertake to actively provide such counseling to members of their local community whom they have access to. Last, but certainly not the least, manufacturers of EO-based products should voluntarily and routinely incorporate prominent hazard warnings, preferably pictorial/symbolic ones, on product labels and packages.

## Conclusions

In this case of accidental EO ingestion by a 17-month-old female infant, the child was found to be drowsy at the time of admission to the hospital. However, she did not exhibit any other symptoms like vomiting or seizures that are also associated with EO ingestion. Two stomach washes with 50 mL normal saline were carried out. This was followed by the oral administration of 25 mg ranitidine as an anti-emetic to prevent dehydration due to vomiting episodes. Thereafter, only supportive care was given. The patient made a complete recovery over 24 hours and there was no recurrence of symptoms. 
